# Supportive couple relationships buffer against the harms of HIV stigma on HIV treatment adherence

**DOI:** 10.1186/s12889-023-16762-w

**Published:** 2023-09-28

**Authors:** Sarah A. Gutin, Allison Ruark, Lynae A. Darbes, Torsten B. Neilands, James Mkandawire, Amy A. Conroy

**Affiliations:** 1 Department Of Community Health Systems, School of Nursing, University of California, San Francisco (UCSF), 2 Koret Way, San Francisco, CA 94143 USA; 2 Wheaton College, 501 College Avenue, Wheaton, IL USA; 3 Department of Health Behavior and Biological Sciences, School of Nursing, University of Michigan, 400 North Ingalls Building, Ann Arbor, MI USA; 4 Division of Prevention Sciences, University of California, San Francisco (UCSF), 550 16Th. Street, #3311, San Francisco, CA 94158 USA; 5 Invest in Knowledge, Old Naisi Road, P.O. Box 506, Zomba, Malawi

**Keywords:** Anticipated HIV stigma, Heterosexual couples, Antiretroviral therapy, Relationship dynamics, Sub-Saharan Africa

## Abstract

**Introduction:**

HIV stigma can impact couple relationships through stress or bring partners closer through shared experiences. Conversely, couple relationships may protect against the harms of stigma, including anticipated stigma on negative health outcomes. Yet few studies have assessed the potential link between HIV stigma, relationship dynamics, and antiretroviral therapy (ART) adherence. Using dyadic data from a cross-sectional study of Malawian couples living with HIV, we tested associations between anticipated stigma and: 1) relationship dynamics (e.g., trust, sexual satisfaction, communication) and partner support; and 2) self-reported ART adherence.

**Methods:**

Heterosexual couples (211 couples, 422 individuals) with at least one partner on ART were recruited from clinics in Zomba, Malawi. Partners completed separate surveys on anticipated stigma, relationship dynamics, and ART adherence. Linear mixed models evaluated associations between anticipated stigma and relationship dynamics, and whether associations varied by gender. Generalized estimating equation models tested for associations between anticipated stigma and high ART adherence (90–100% vs. < 90%) at the individual level, and whether they were moderated by relationship dynamics at the couple level.

**Results:**

Couples' relationship length averaged 12.5 years, 66.8% were HIV sero-concordant, and 95.6% reported high ART adherence. In multivariable models, sexual satisfaction (β = -0.22, 95%CI = -0.41;-0.03, *p* = 0.020) and partner social support (β = -0.02, 95%CI = -0.04;-0.01, *p* < 0.01) were negatively associated with anticipated stigma. Significant interaction effects showed that adherence is moderated in couples with higher partner support and sexual satisfaction such that adherence is lowest when anticipated stigma is high and social support is low, and that adherence is lowest when anticipated stigma is high and sexual satisfaction is low.

**Conclusions:**

Increased anticipated stigma is most associated with lower ART non-adherence at lower levels of social support and sexual satisfaction. Conversely, supportive and fulfilling relationships may buffer the negative association between stigma and ART adherence. Couples’ interventions that focus on improving communication and support systems within couples could reduce the negative impacts of anticipated stigma on couples living with HIV.

## Introduction

Despite global efforts to fight HIV stigma and increase access to care and treatment, HIV stigma remains a major obstacle to ending the AIDS epidemic by 2030 [1]. HIV stigma limits access to healthcare, economic, and social resources that are needed to live a healthy life with HIV [1–3]. Stigma refers to a social process that can lead to the exclusion of individuals or groups based on real or perceived characteristics [4]. Globally, HIV stigma remains a significant barrier to HIV testing [5–7], linkage to care and treatment, and adherence to antiretroviral therapy (ART) [1, 8–10]. Experiences of HIV stigma are also linked to non-disclosure of HIV status [11, 12], condomless sex [13], and unsuppressed viral load [14–16], which can result in transmission of HIV to sexual partners [17].

Stigma occurs at multiple levels, from internalized stigma to stigma experienced in interpersonal relationships, at the couple level, and through institutions and social structures. HIV stigma has been grouped into four main domains in which people internalize real or perceived stigma onto themselves (internalized stigma), experience overt acts of discrimination (enacted stigma), anticipate or fear enacted stigma (anticipated stigma), and perceive how others view and treat people living with HIV (perceived stigma) [1]. Research suggests that when a partner first tests positive, there is more intra-dyadic stigma and concerns about rejection, discrimination, abandonment, and violence [18–21], but this may decline over time, with the primary source of HIV stigma coming from outside the couple relationship [5, 22–25]. According to couple interdependence theory [26], both partners’ beliefs, experiences, and behaviors impact the other partner’s beliefs, experiences, and behaviors regardless of which partner lives with HIV. This may be particularly relevant in settings such as Malawi where married individuals are viewed as a single marital body with shared characteristics [27, 28]. In South Africa, a clinical trial of an HIV-stigma intervention with pregnant women living with HIV and their partners found that women’s reports of HIV stigma in the control arm were correlated with male partners’ stigma scores among men living with HIV, suggesting that stigma can act interdependently in couples and is not an isolated issue for only one partner [29]. Therefore, interventions are needed that go beyond the individual level to focus on interpersonal and other levels where stigma occurs and can negatively impact health.

HIV-related social support from primary partners may reduce or buffer the harms of anticipated stigma. Studies have highlighted the positive role of partner support on health-enhancing behaviors, such as HIV testing, HIV status disclosure, and ART adherence [27, 30–33]. However, little research has been conducted on how anticipated stigma impacts couple relationships, and how relationship dynamics (e.g., intimacy, partner support) in turn affect stigma. Previous research has focused on the negative effects of HIV stigma on non-disclosure to partners [34–37] rather than examining how stigma impacts couples who have already disclosed and should be engaged in HIV care and treatment. Few, if any studies, in sub-Saharan Africa have examined whether relationship dynamics can buffer the negative impacts of stigma on ART adherence.

While supportive relationships may help offset negative HIV stigma effects, it is also possible that HIV stigma could worsen relationship dynamics and damage the couple relationship. Qualitative research found that HIV stigma could damage couple communication regarding HIV and negatively impact sexuality and sexual satisfaction [38, 39]. Stigma from a partner could also increase self-stigmatization and lead to the experience of more HIV stigma [18]. Couple characteristics such as relationship duration and couple HIV serostatus could moderate the association between experiences of HIV stigma and relationship functioning and partner support [40]: if both partners are living with HIV and report experiencing stigma, they may offer greater mutual support and be united by negative experiences as compared to couples in which only one partner is living with HIV. Based on this research [18, 38–40], we hypothesize that the strength and direction of the association between stigma and relationship dynamics may vary by couple serostatus.

Few studies have considered the role of relationship dynamics and social support in studies of HIV stigma in couples. A review paper found that only one quantitative study that examined HIV stigma with couple-level outcomes [41]. Among Chinese sero-discordant couples, individuals with a more couple-centric versus individual-centric orientation reported fewer depressive symptoms, but the protective effect of being in a couple diminished when HIV stigma levels were high [42]. We are not aware of studies that have tested for associations between HIV stigma and relationship dynamics in couples in which one or both partners are living with HIV.

To fill this research gap, we investigated the association between HIV stigma, relationships dynamics, and ART adherence among heterosexual sero-concordant and discordant couples in Malawi. Specifically, we tested for associations between anticipated stigma and relationship dynamics, including social support and couple communication, and whether these associations differed by couple serostatus. Next, we tested for associations between anticipated stigma and ART adherence and whether these associations differed by the same relationship dynamics (e.g., intimacy, partner support, couple communication). This allowed us to test the hypothesis that supportive couple relationships may offset the negative impacts of anticipated stigma on engagement in HIV care. If shown to be associated, this study would provide critical evidence to support interventions that strengthen partner support for couples affected by HIV and build sources of resiliency within couples to fight stigma, improve treatment outcomes, and end AIDS by 2030 [1].

## Methods

### Study context

This study took place in the Zomba district of Southern Malawi, which has an HIV prevalence of approximately 15% [43]. With a well-established ART program, Malawi has over 850,000 individuals on ART [44]. Since the start of a universal test-and-treat approach in 2016 which offers free HIV care and treatment, almost 90% of people living with HIV (PLHIV) in Malawi have started ART [45]. Most adults in Malawi are married or in cohabitating unions [43].

### Study procedures

Data are from the *Umodzi M’Banja* (Unity in the Family) study, a mixed-methods dyadic investigation of PLHIV and their primary partners in Zomba, Malawi [46–48]. We conducted a cross-sectional survey with 211 couples (422 individuals) from August to November 2017. Couples were eligible to participate if they were: (1) age 18 + ; (2) in non-polygamous married or cohabitating unions for at least six months; and (3) had at least one partner (referred to as the “index patient”) on ART for two months or more, who had disclosed their HIV status to their primary partner.

Participants were recruited at two high-volume HIV clinics, a private clinic at a rural community hospital and an urban clinic at a large district hospital acting as a regional referral center. Research staff announced the study in waiting rooms during daily health talks and interested index patients could approach the staff for more information. If the index patient was eligible, they were given an information card to share with their primary partner who could contact study staff for screening. Partner eligibility was assessed over the phone and confirmed in-person at an interview appointment with the couple.

Partners were consented separately in private locations at the HIV clinics and were both provided with a small incentive (around $2 USD) for their time. Gender-matched research assistants administered surveys using tablet devices that housed a secure, web-based data collection platform. Surveys were translated into the local language, back-translated into English by an independent person, and then administered in Chichewa. Partners were interviewed separately, but simultaneously, in private spaces at the HIV clinics, and were asked questions on relationship dynamics, partner support, and if they were living with HIV, and about anticipated stigma and ART adherence.

### Measures

We examined two dependent variables (anticipated HIV stigma and self-reported ART adherence) and several independent variables of interest. In the first set of models, anticipated HIV stigma was treated as a dependent variable (continuous) and we examined associations with various relationship dynamics (including continuous variables for sexual satisfaction, relationship intimacy, trust, equality, unity/ “we-ness”, and commitment), with partner support (continuous variables for general partner support and HIV treatment-specific partner support), and with couple communication patterns (including continuous variables for withdrawal, demanding, and avoidant communication styles). In a second set of models, self-reported ART adherence was the dependent variable (binary, adherent vs. non-adherent) and the independent variables of interest included anticipated HIV stigma (continuous) with two potential moderators, general partner social support (continuous), and sexual satisfaction (continuous). All study measures are summarized in Table 1.

**Table 1:** Measures used in the Umodzi M’Banja Study in Malawi

Category	Construct	Scale	Sample question	Number of Items	Response options	Cronbach’s alpha (α)	Scale range	Male Mean score (SD)	Female Mean score (SD)
Stigma	Anticipated HIV stigma	Anticipated stigma scale	“Family members will use my HIV status against me”	8 items	1 = Highly unlikely 2 = Unlikely 3 = Neutral 4 = Likely 5 = Highly likely	0.89	1—5	1.70 (0.79)	1.58 (0.70)
Relationship dynamics	Sexual satisfaction	General ﻿Couple Sexual Satisfaction Scale (CSSS-Gen)	“I am satisfied with the frequency with which I have sexual intercourse”	13 items	1 (strongly disagree) to 5 (strongly agree)	0.90	1—5	4.28 (0.61)	4.50 (0.58)
	Relationship intimacy	Triangular Scale of Love	“I have a relationship of mutual understanding with my partner”	5 items	1 (strongly disagree) to 5 (strongly agree)	0.90	1—5	4.54 (0.62)	4.51 (0.65)
	Trust	Dyadic trust scale	“My partner is honest with me”	8 items	1 (strongly disagree) to 5 (strongly agree)	0.82	8—40	32.92 (4.85)	32.20 (6.65)
	Relationship equality	intimacy subscale of the Relationship Values Scale	“My partner and I have equal power in the relationship”	8 items	1 (strongly disagree) to 5 (strongly agree)	0.87	8—40	35.34 (4.87)	33.19 (5.73)
	Unity / “we-ness”	inclusion-of-other-in-self diagram	Visual scale	1 item	1 (no overlap) to 7 (complete overlap)	_	1—7	6.61 (0.92)	6.01 (1.25)
	Relationship commitment	Triangular Scale of Love	“I am committed to maintaining my relationship with my partner”	8 items	1 (strongly disagree) to 5 (strongly agree)	0.82	1—5	4.52 (0.55)	4.64 (0.40)
Partner support	General partner support	Subscales from the Social Provisions Scale (partner version)	“I can depend on my partner to help me if I really need it”	12 items	1 (strongly disagree) to 5 (strongly agree)	0.88	12—60	52.16 (6.89)	51.80 (8.62)
	HIV treatment-specific partner support	SPS scale	“I can depend on my partner to help me with my antiretrovirals if I really need it”	9 items	1 (strongly disagree) to 5 (strongly agree)	0.84	9 – 45	38.78 (7.80)	39.78 (6.18)
Couple communication	Avoidant communication	Communications Pattern Questionnaire	“When an issue or problem arises, both of us avoid discussing the problem”	2 items	1 (very unlikely) to 5 (very likely)	0.75	1—5	1.8 (0.87)	2.40 (1.08)
	Withdrawal communication			3 items			1 – 5	2.32 (1.01)	2.46 (1.06)
	Demanding communication			3 items			1—5	2.38 (1.02)	2.61 (1.08)

#### Anticipated HIV stigma

Anticipated HIV stigma was measured with the anticipated HIV stigma scale [49]. This scale captures future expectations of stereotyping, discrimination, and/or prejudice from family members and providers because of one’s HIV status. Only participants who were living with HIV were asked these questions. Therefore, if both members of the couple were living with HIV, they both reported on their own anticipation of stigma. However, if the couple was sero-discordant, then only the PLHIV reported on anticipated stigma. This scale has been previously validated in sub-Saharan Africa with PLHIV [50–52]. Response options ranged from 1 (highly unlikely) to 5 (highly likely) (Table 1 for details). A mean of stigma items was calculated with higher scores across a range from 1 to 5 indicating higher anticipated stigma. In all analyses, the scale was used as a continuous variable.

#### Relationship dynamics

We assessed relationship dynamics by measuring sexual satisfaction, relationship intimacy, trust, equality, unity/ “we-ness”, and commitment (Table 1). Row means or row totals were created for each scale based on the original scoring procedures (see Table 1 for the ranges for each variable). Couple-level variables were created such that each represented the couple-level mean score from both partners.

Sexual satisfaction was measured using the General Couple Sexual Satisfaction Scale (CSSS-Gen) [53], which was validated in Malawi. Relationship intimacy was measured using a subscale from the Triangular Scale of Love [54]. We used the shortened version of this scale validated in Malawi [55]. Trust was measured with the Dyadic Trust Scale [56] which has been validated in various sub-Saharan African countries [57–59]. Relationship equality was measured with the intimacy subscale of the Relationship Values Scale [60] which was previously validated in another Malawian study [53]. Unity or “we-ness” was measured with a single item using the inclusion-of-other-in-self diagram [61]. The diagram asks respondents to pick from a set of over-lapping circles that best describes their relationship with their partner. Response options included seven sets of circles that ranged from 1 (no overlap) to 7 (complete overlap) with higher scores indicating greater relationship unity. This scale was previously validated in Malawi [53]. Relationship commitment was measured with a subscale from the Triangular Scale of Love [54], which was previously validated in Malawi [55].

#### Partner support

Partner support was measured in two ways (Table 1). We assessed general partner support with three subscales from the Social Provisions Scale (partner version) representing guidance, attachment, and reliable assistance [62]. These items were selected because they capture aspects of emotional, instrumental, and informational support that were salient in previous studies in Malawi and South Africa [27, 33]. We also developed a measure of HIV treatment-specific partner support that was adapted from the SPS scale [63] and validated in Malawi [53]. Both partner support variables were treated as continuous variables in all models.

#### Couple communication patterns

Couple communication patterns (i.e., engaging in withdrawal, demanding, or avoidant communication styles) were measured with an adapted version of the Communications Pattern Questionnaire [64]. This scale has been previously validated in Malawi [55]. Variables for withdrawal, demanding, and avoidant communication styles were all treated as continuous variables in models.

#### Self-reported adherence to ART

To account for low educational attainment, adherence was assessed using the “bean method” for low literacy populations [46] based on the 30-day Visual Analog Scale [65]. The interviewer gave the participant two bowls, one with beans and one empty, and explained that the beans represent the ART that they take each month. Participants were instructed to select the number of beans corresponding to the pills they did not take in the last month and put them in the second bowl. A binary variable was created based on treatment regimen (once or twice per day) and the number of beans selected. We considered taking 90% or more of pills to be adherent and less than 90% to be non-adherent [65]. The 90% cutoff was chosen because while ≥ 95% adherence is considered perfect or near perfect adherence, prior research has shown that with newer ART regimens, HIV viral suppression for persons with 90–94% adherence did not differ from those with ≥ 95% adherence [66, 67]. The 90% adherence cutoff is also a validated cutoff used in other studies in SSA [68]

#### Covariates

Based on the previous literature on couples and HIV [46, 69], multivariable models controlled for age (continuous), gender, years of education (continuous), couple HIV status (concordant or discordant), relationship duration (continuous), and household wealth score (continuous), which is a proxy for socio-economic status [70].

### Data analysis

One-way frequency tables and measures of central tendency were generated to characterize the sample. Linear mixed models [71] tested for associations between relationship dynamics (independent variables) and anticipated stigma (dependent variable) and whether this association varied by couple serostatus, after controlling for socio-demographics (gender, age, education, household wealth score) and relationship characteristics (relationship duration, couple sero-status), in accordance with literature on HIV risk behaviors and relationship dynamics [72]. Models also adjusted for clustering at the couple-level by including a random intercept for the couple to control for non-independence of responses from individuals within the same couple who both reported on the outcome of anticipated stigma. In addition, models included the cluster-robust standard error option [73–75] to protect inferences against normality and homoskedasticity assumption violations.

In addition, using generalized estimating equation (GEE) models clustering at the couple-level with the robust standard error option [73–75], a binary distribution, and a logit link function to yield odds ratios [76], we tested for associations between anticipated stigma and ART adherence after controlling for socio-demographics and relationship duration. Using ad hoc analyses, we also examined whether this association was moderated by relationship dynamics (e.g., intimacy, trust) and partner support by including interaction terms in the models. To evaluate whether associations differed by couple sero-status and gender, we included respective interaction terms in the models. Initial models were specified to contain main effects. Models were then extended by adding relevant interaction terms one by one to test the moderation hypotheses described previously. If interactions were significant, results from the models with interaction terms included are reported. We considered an alpha of *p* < 0.05 to be statistically significant. Overall, missing data were negligible (less than 5% on any given variable). All analyses were performed using Stata 16 (College Station, TX).

### Ethical approval

The study was approved by the National Health Science Research Committee in Malawi (IRB # 15/12/1512) and the Human Research Protection Program at the University of California, San Francisco (IRB # 15–17394). Informed consent was obtained for all individual participants included in the study. All procedures followed were in accordance with the Helsinki Declaration of 1975, as revised in 2000.

## Results

### Sample characteristics

Of 422 participants, the mean age was 40.5 years and the majority (80.8%) had a primary education or less (Table 2). All couples were married/cohabitating and had been together on average for 12.5 years. Two-thirds of couples were sero-concordant positive (66.8%). Of participants who were living with HIV (*N* = 352), the majority were on ART (82.5%) and 95.6% reported 90–100% ART adherence in the past 30 days. The mean anticipated stigma score was 1.6 (scale range 1–5).

**Table 2:** Sample characteristics of couples living with HIV in the Umodzi M’Banja Study, Malawi (211 couples; 422 individuals)

Variable	Total sample %, Mean (SD)	Men %, Mean (SD)	Women %, Mean (SD)	t / Pearson χ^2^	DF	*p*-value for gender differences
***Individual characteristics***						
Age (years)	40.5 (10.2)	43.5 (10.6)	37.4 (8.8)	-6.4388	419	< 0.001
Education level						
Primary school or less	80.8	73.5	88.2	15.4638	2	< 0.001
Secondary school	18.7	25.6	11.8			
Tertiary school or higher	0.5	1.0	0.0			
Household wealth index (range: 0–8)	2.8 (1.6)	3.0 (1.6)	2.7 (1.6)	-2.0337	413	0.04
Religion						
Muslim	10.2	10.0	10.4	0.0209	1	0.89
Christian	89.8	90.0	89.6			
Living with HIV	83.4	81.0	85.8	2.4580	2	0.29
Currently on ART	82.5	79.6	85.3	2.3597	1	0.13
Length of time on ART (years)	4.8 (3.1)	4.6 (3.1)	5.1 (3.1)	1.6023	346	0.11
90–100% adherence reported^a^	95.6	96.4	94.8	0.4725	1	0.49
Anticipated stigma score (range 1–5)	1.6 (0.74)	1.70 (0.1)	1.58 (0.1)	-1.4280	349	0.15
***Relationship characteristics***						
Relationship duration (years)	12.5 (9.0)	12.9 (9.2)	12.1 (8.8)	-0.9673	418	0.33
Number of dependent children (range: 0–11)	3.3 (1.8)	3.5 (1.9)	3.2 (1.7)	-1.3595	420	0.17
Sero-concordant positive	66.8	–	–			

^a^ART adherence is presented as a binary variable where taking 90% or more of pills was considered to be adherent and less than 90% to be non-adherent

We tested whether anticipated stigma scores were higher for sero-discordant versus sero-concordant couples. We found higher levels for sero-discordant couples, but the difference was not statistically significant (*p* = 0.345). In addition, we tested to see if stigma scores within couples were correlated for sero-concordant couples. However, we only found a small correlation between partners’ stigma scores (*r* = 0.28).

### Associations between relationship factors and anticipated stigma

In multivariable models, sexual satisfaction was significantly associated with anticipated stigma such that HIV-positive individuals in a relationship with higher sexual satisfaction reported lower levels of anticipated stigma (β = -0.22, 95%CI = -0.41;-0.03, *p* = 0.020) (Table 3). Associations did not vary by couple serostatus or gender. Other relationship dynamics such as intimacy, trust, equality, “we-ness”/unity, and commitment did not show significant associations with stigma.

**Table 3:** Associations between relationship dynamics and HIV stigma among persons living with HIV in Malawi (*N* = 345)

Variable	Unadjusted Beta coefficient (β)	95% CI	*P*-value	Adjusted Beta coefficient (β)	95% CI	*P*-value
Intimacy	-0.10	-0.26, 0.07	0.257	-0.13	-0.31, 0.55	0.172
Trust	-0.01	-0.03, 0.01	0.425	-0.01	-0.03, 0.01	0.209
Equality	-0.01	-0.03, 0.01	0.244	-0.02	-0.04, 0.01	0.164
“We-ness”/unity	-0.04	-0.16, 0.08	0.520	-0.06	-0.18, 0.07	0.374
Sexual satisfaction	-0.19	-0.36, -0.01	0.042	-0.22	-0.41, -0.03	**0.020**
Commitment	-0.08	-0.32, 0.17	0.537	-0.11	-0.37, 0.15	0.414
**Partner support (general)**	-0.02	-0.03,-0.004	0.014	-0.02	-0.04, -0.01	**0.006**
**Partner support (HIV treatment-specific)**	-0.01	-0.03, 0.003	0.102	-0.02	-0.04, -0.0003	**0.046**
**Communication style**						
Withdrawal	0.12	0.04, 0.20	0.004	0.13	0.04, 0.21	**0.003**
Demanding	0.15	0.08, 0.23	< 0.001	0.17	0.09, 0.24	**< 0.001**
Avoidant	0.26	0.13, 0.38	< 0.001	0.26	0.13, 0.39	**< 0.001**

Multivariable models controlled for gender, age, years of education, relationship duration, household wealth, and couple HIV status. All relationship dynamics variables listed are continuous variables

In the multivariable models on partner support, both general partner support (β = -0.02, 95%CI = -0.04;-0.01, *p* = 0.006) and HIV treatment-specific partner support (β = -0.02, 95%CI = -0.04;-0.0003, *p* = 0.046) were associated with less anticipated stigma. In the multivariable models on communication, negative communication styles such as withdrawal (β = 0.13, 95%CI = 0.04;0.21, *p* = 0.003), demanding (β = 0.17, 95%CI = 0.09;0.24, *p* < 0.001), and avoidant communication (β = 0.26, 95%CI = 0.13;0.39, *p* < 0.001) were associated with higher stigma (Table 3). Associations did not vary by couple serostatus or by gender.

### Associations between anticipated stigma and ART adherence

In multivariable models on ART adherence, the odds of having high adherence were 45% lower for each one-unit increase in anticipated stigma (aOR = 0.55, 95%CI = 0.34;0.89, *p* = 0.014) (Table 4). A one-unit increase corresponds to a participant saying stigma is “likely” vs. “highly likely”. Given the significant associations described above, we also tested whether there were any interactions between partner social support and anticipated stigma and between sexual satisfaction and anticipated stigma and associations with ART adherence. The models showed significant positive interactions between partner social support and anticipated stigma (aOR = 1.10, 95%CI = 1.01; 1.20, *p* = 0.032) and sexual satisfaction and anticipated stigma (aOR = 3.25, 95%CI = 1.06; 9.93, *p* = 0.039) such that the association between higher stigma and non-adherence was moderated in couples with higher levels of partner social support and sexual satisfaction (Table 4). Associations did not vary by couple serostatus or gender.

**Table 4:** Adjusted odds ratios (aORs) for associations between HIV stigma and ART adherence among couples living with HIV in Malawi (*n* = 337)

	**Model 1: Association of Stigma on ART adherence**		**Model 2: Association of social support x stigma on ART adherence**		**Model 3: Association of sexual satisfaction x stigma on ART adherence**	
**Variable**	**aOR (95% CI)**	***p***	**aOR (95% CI)**	***p***	**aOR (95% CI)**	***p***
HIV stigma	0.55 (0.34; 0.89)	**0.014**	0.01 (0.00; 0.45)	**0.020**	0.003 (0.00; 0.48)	**0.025**
Couple HIV status	0.31 (0.11; 0.89)	**0.029**				
Social support			0.93 (0.76; 1.13)	0.451		
Social support x stigma interaction			1.10 (1.01; 1.20)	**0.032**		
Sexual satisfaction					0.29 (0.03; 2.96)	0.294
Sexual satisfaction x stigma interaction					3.25 (1.06; 9.93)	**0.039**

Multivariable models controlled for gender, age, years of education, relationship duration, and couple HIV status. HIV stigma, social support, and sexual satisfaction variables were all continuous

**Fig. 1:**
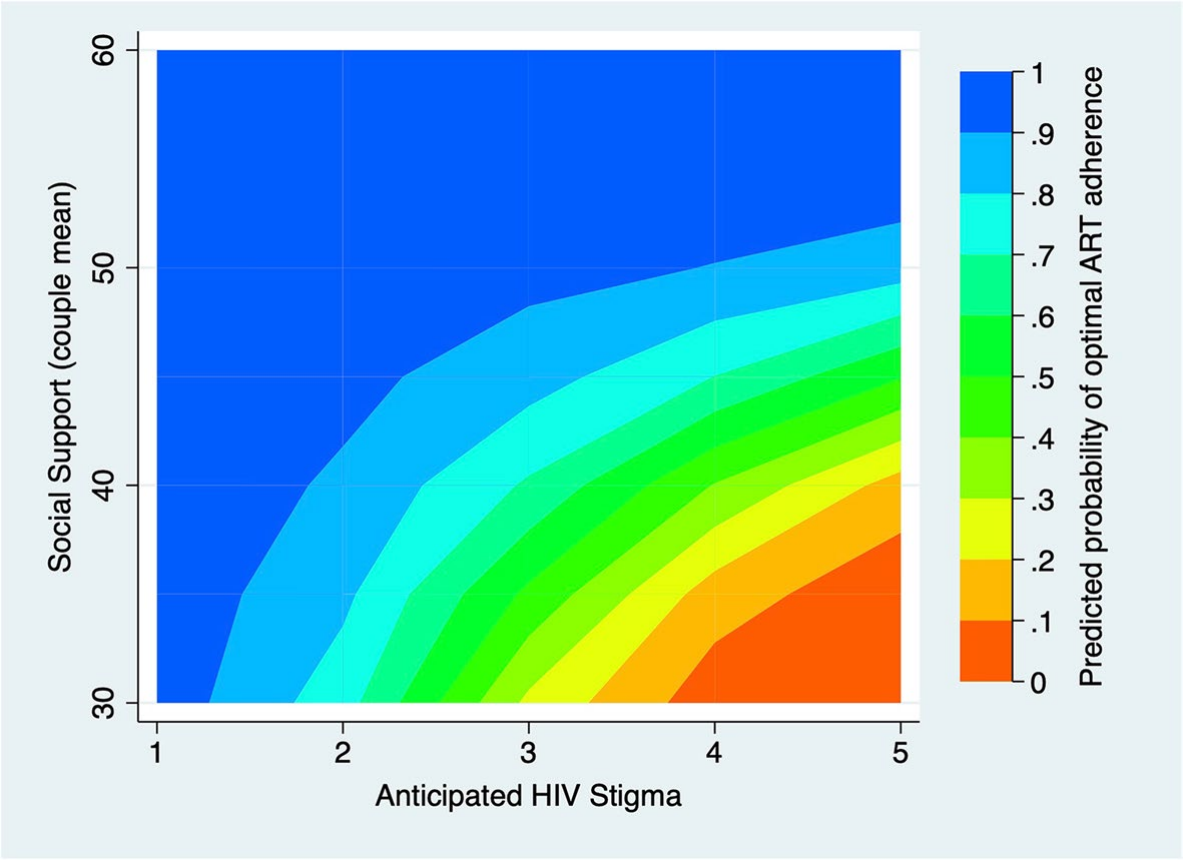
The predicted probability of optimal ART adherence at different levels of anticipated HIV stigma and social support

**Fig. 2:**
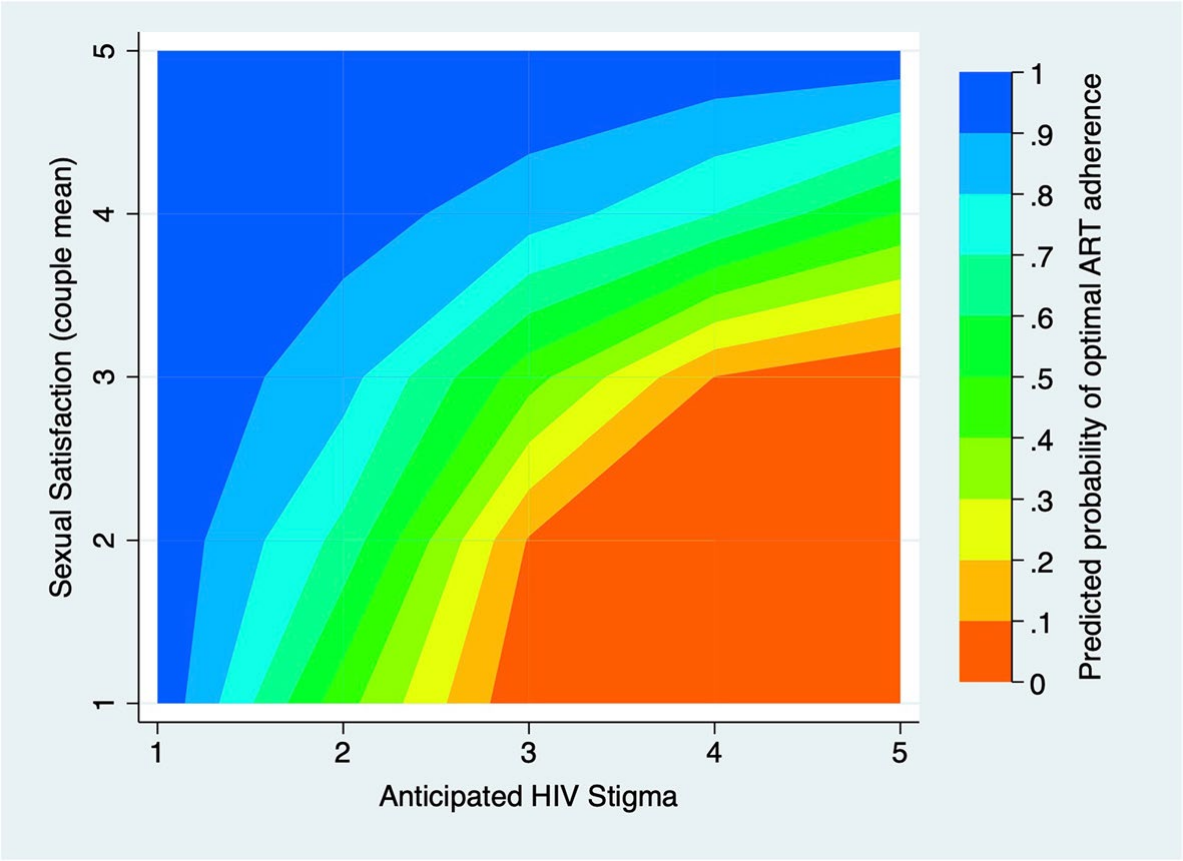
The predicted probability of optimal ART adherence at different levels of anticipated stigma and sexual satisfaction

To aid in understanding these interaction terms, we developed two contour plots [77, 78] that examine 1) the predicted probability of optimal ART adherence at different levels of anticipated stigma and social support (Fig. 1), and 2) the predicted probability of optimal ART adherence at different levels of anticipated stigma and sexual satisfaction (Fig. 2). In Fig. 1, adherence is lowest when anticipated stigma is high and social support is low (dark orange region, lower right corner) whereas adherence is highest when social support is high or anticipated stigma is low (or both are true) as represented by the blue region (upper left corner). In Fig. 2, we see that adherence is lowest when anticipated stigma is moderate to high and sexual satisfaction is low to moderate (dark orange region, lower right corner) whereas adherence is highest when sexual satisfaction is high or anticipated stigma is low (or both are true) as represented by the dark blue region (upper left corner).

## Discussion

Among couples in Malawi, we examined associations between relationship dynamics (relationship quality, partner support, and communication) and anticipated HIV stigma, as well as the association of anticipated stigma with ART adherence. We found that higher sexual satisfaction and partner social support were associated with less anticipated stigma, and that negative communication styles were associated with higher anticipated stigma. In addition, significant interaction effects showed that the association between higher stigma and suboptimal adherence was moderated in couples with higher partner support and sexual satisfaction. This is one of the first studies to examine the role that relationship dynamics and social support may play in mitigating anticipated stigma in couples and whether relationship dynamics can buffer the negative impacts of stigma on ART adherence. Insights from this study can help researchers develop couple-based interventions to improve important aspects of relationships and build couple resiliency that may lessen the negative impact of anticipated stigma on HIV treatment outcomes.

In our study, individuals with higher levels of sexual satisfaction reported lower levels of anticipated stigma, whereas other relationship quality constructs such as intimacy, trust, unity, and equality did not show this association. Previous research has highlighted the importance of sexual satisfaction in relationship quality [79] and relationship satisfaction [80, 81] and its impact on sexual and reproductive health [55, 82, 83]. It may be that couples who have a fulfilling sex life anticipate less stigma from outside their relationships. Some PLHIV have reported that HIV limits or reduces sexual intimacy [84–86]. Thus, couples who report high sexual satisfaction despite living with HIV may have particularly healthy and resilient relationships, which also provide a buffer against stigma. Couple-based interventions that promote a healthy sex life, and normalize sex and HIV, may help protect couples from the experience of stigma.

In addition, our findings highlight the importance of partner support and communication for anticipated stigma. Couples with more supportive relationships experienced lower anticipated stigma. Also, the association between higher stigma and lower adherence was moderated in couples with higher social support and sexual satisfaction. It may be that people who are in strong, healthy relationships may feel more secure and comfortable with HIV as well as anticipate less stigmatization and discrimination. Finally, negative communication styles were associated with higher anticipated stigma. Partner support and positive couple communication have been linked to positive HIV-related health behaviors such as uptake of couple HIV counselling and testing [87, 88], HIV status disclosure [30], encouraging partner ART use [89], and adherence to ART more broadly [33, 46, 89]. Thus, couples’ interventions focused on helping couples develop skills such as healthy couple communication and providing partner support may be optimal for reducing stigma and helping overcome barriers to ART adherence that arise from stigma and discrimination outside the relationship [18, 25, 90].

We also found that being in a sero-discordant relationship did not weaken the potential protective effect of relationship factors on anticipated stigma, meaning that the association does not depend on couple serostatus. This could suggest that HIV is becoming more normalized with widespread access to ART and “undetectable equals untransmittable” messaging, and that sero-discordant couples who have disclosed are as resistant to stigma as those who are sero-concordant. Recent studies have found that partners are providing an elevated role in offering HIV-related support [89] and there are also signs of growing resistance to stigma from outside the couple, which may be levelling the playing field for discordant and concordant couples [3].

A study strength was that we could report on perspectives of both partners and analyze the dyad as a unit, thus adding to previous research that has examined anticipated stigma at the individual level. By incorporating both partners’ perspectives of relationship dynamics, we can overcome potential biases that might be present if just one partner was reporting on their relationship. A possible limitation is that study couples may have more positive relationship dynamics and less anticipated stigma than the general population, given that both partners decided to enroll in a couples’ study. Thus, these findings may not be fully generalizable to other populations that may have higher levels of stigma and poorer relationship dynamics. Other characteristics of study participants may also reduce the generalizability of our findings. For example, the mean age in our sample was 40 years, couples had been together for many years (approximately 14 years), and almost 96% reported optimal adherence. Our results may best represent older, established couples who are better engaged in care, as opposed to younger couples who may face greater challenges with adherence and stigma. We also do not know the HIV status of couples at the time of marriage, and so we cannot speculate as to whether people were trying to sero-sort or choose partners based on their HIV status. Future studies that follow couples at the start of the partnership could help to disentangle the effects of stigma over the course of the relationship. In addition, all measures, including adherence measures, were self-reported and may be affected by social desirability bias. Finally, as this was a cross sectional study, we cannot establish causality. Longitudinal research would be needed to explore how relationship dynamics and anticipated stigma impact adherence to ART over time among couples living with HIV. Qualitative studies that explore the nature of stigma in dyads would also add nuance to this topic.

## Conclusions

This research suggests that couple relationships could be leveraged as an important source of resilience and support. Interventions that build resiliency in couples and strengthen couple relationships, with a focus on constructive forms of communication, building emotional and practical support within couples, and a healthy sexual life, could reduce the negative impact of extra-dyadic HIV stigma on the health of couples living with HIV in sub-Saharan Africa. Such interventions could have a positive impact on HIV care outcomes, such as ART adherence, which can help to attain population-level goals for reducing new HIV infections. In over 40 years of the HIV epidemic, not enough progress has been made on eliminating HIV stigma by focusing on individuals in isolation of their social environment. By working with both partners together to fight HIV stigma, we can target the experience of stigma within the dyad while also addressing societal and structural stigma by building couple resiliency.

## Data Availability

The datasets used and/or analyzed during the current study are available from the corresponding author on reasonable request.

## Abbreviations

ART: Antiretroviral therapy
CSSS-Gen: General Couple Sexual Satisfaction Scale
GEE: Generalized estimating equation
HIV: Human immunodeficiency virus
PLHIV: People living with HIV
